# Predicting the outcome of conservative treatment with physiotherapy in adults with shoulder pain associated with partial-thickness rotator cuff tears – a prognostic model development study

**DOI:** 10.1186/s12891-018-2239-8

**Published:** 2018-09-11

**Authors:** Cordula Braun, Nigel C. Hanchard, Helen H. Handoll, Andreas Betthäuser

**Affiliations:** 1 School of Health and Social Care, Teesside University, Middlesbrough, UK; 2 Present address: Faculty of Health and Physiotherapy, Buxtehude, Germany; 3 schulter-zentrum.com, Hamburg, Germany; 4 Faculty of Health, Harburger Str. 6, 21614 Buxtehude, Germany

**Keywords:** Shoulder pain, Rotator cuff, Conservative treatment, Physical therapy, Prognosis, Prognostic model development

## Abstract

**Background:**

Rotator cuff disorders represent the commonest type of painful shoulder complaints in clinical practice. Although conservative treatment including physiotherapy is generally recommended as first-line treatment, little is known about the precise treatment indications for subgroups of rotator cuff disorders, particularly people with shoulder pain associated with partial-thickness tears of the rotator cuff, PTTs: “symptomatic PPTs”. The aim of this study was to develop a prognostic model for predicting the outcome of a phase of conservative treatment primarily with physiotherapy in adults with symptomatic PTTs.

**Methods:**

A prospective observational cohort study was conducted in an outpatient setting in Germany. Ten baseline factors were selected to evaluate nine pre-defined multivariable candidate prognostic models (each including between two and nine factors) in a cohort of adults with symptomatic atraumatic PTTs undergoing a three-month phase of conservative treatment primarily with physiotherapy. The primary outcome was change in the Western Ontario Rotator Cuff Index. The models were developed using linear regression and an information-theoretic analysis approach: Akaike’s Information Criterion (AIC_C_).

**Results:**

Eight candidate models were analyzed using data from 61 participants. Two “best models” were identified: smoking & pain catastrophizing and disability & pain catastrophizing. However, none of the models had a satisfactory performance or precision.

**Conclusions:**

We could not determine a prognostic model with satisfactory performance and precision. Further high-quality prognostic model studies with larger samples are needed, but should be underpinned, and thus preceded, by robust research that enhances knowledge of relevant prognostic factors.

**Study registration:**

DRKS00004462. Registered 08 April 2014; retrospectively registered (prior to the analysis).

**Electronic supplementary material:**

The online version of this article (10.1186/s12891-018-2239-8) contains supplementary material, which is available to authorized users.

## Background

Painful shoulder complaints are common musculoskeletal disorders in clinical practice [1], most being attributed to rotator cuff pathology [2, 3]. Rotator cuff pathology encompasses a range of pathologies from tendinopathy to tears, which may be partial- or full-thickness [4]. Reported rates of symptomatic partial-thickness tears (PTTs), the condition of interest in this study, vary between 7% [5] and 24% [6] in shoulder pain populations. Of the four rotator cuff tendons (supraspinatus, infraspinatus, teres minor, subscapularis), the supraspinatus is by far the most often affected [7], and also usually the first to tear [8, 9]. In order to concisely label the population of interest, we use the term “symptomatic PTT” to describe people with shoulder pain in the presence of a PTT of the rotator cuff.

The clinical presentation of symptomatic PTTs is essentially that of “shoulder impingement” [7, 9, 10]. Verification of a PTT requires diagnostic imaging, commonly ultrasonography (US) or magnetic resonance imaging (MRI) [11].

Current guidelines for rotator cuff disorders [12, 13] recommend conservative treatment with medical care and physiotherapy as the first-line treatment; surgical intervention being mainly reserved for non-responders. Head-to-head comparisons of conservative and surgical interventions [14] have overall shown no clinically relevant differences. However, utilisation of surgery for rotator cuff disorders has significantly increased in many countries [15–17], with physiotherapy bypassed in some cases [18]. Both unnecessary surgery and ineffective conservative treatment are undesirable. Knowledge about a patient’s likely response to conservative treatment at the point of diagnosis would save time, effort and suffering, limit exposure to the risks of surgery, and inform distribution of resources. “Understanding which patients [with rotator cuff tears] do best with non-operative treatment” has been rated a top “priority scientific research issue” ([19], p. 10).

The importance of predicting individuals’ responses to particular interventions is increasingly recognized [20], with a corresponding development in prognosis research methodology [21, 22]. One aspect of prognosis research involves the identification of single, independent factors [23]. However, these are unlikely to predict outcomes satisfactorily. Multivariable prognostic models are better placed as they account for real-life clinical complexities [24, 25]. Estimates of prognosis are highly context-dependent, with relevant contextual factors being existing diagnostic and treatment practices, time and place.

Prognostic model research encompasses three key phases: development including internal validation; external validation; and evaluation of clinical impact [25]. External validation is essential before a model may be usable in practice [25]. While prospective cohort studies are generally considered the preferable design for the initial development of a prognostic model [25–27], evaluations of the clinical impact of a prognostic model ultimately require comparative studies.

Our systematic review of the evidence on prognostic models for predicting outcomes in adults undergoing physiotherapy for rotator cuff disorders showed a lack of clinically usable prognostic models and, crucially, of prognostic model research on PTTs [28]. The study’s primary aim was to develop a multivariable prognostic model for the outcome of a phase of conservative treatment with physiotherapy in adults with symptomatic atraumatic PTTs. Secondary aims were to determine the incidence of tear progression and to establish participants’ perceived change of their shoulder complaints over time.

## Methods

The study was based on an a priori protocol and was approved by the Teesside University School of Health and Social Care Research Governance & Ethics Committee and the Ethics Commission of the Hamburg Medical Council (Germany). It was registered in the German Clinical Trials Register (reg.no DRKS00004462). The study design was informed by the most current methodological guidance available at the time of planning [21, 22]. All deviations from protocol were discussed and recorded prior to implementation [29]; the only two relevant deviations are flagged up in this section. This report complies with the items required by the TRIPOD (Transparent Reporting of a multivariable prediction model for Individual Prognosis Or Diagnosis) prediction model development checklist [30].

### Study design, setting and key dates

We conducted a prospective observational single-group cohort study set in Hamburg**,** Germany. All recruitment and assessments took place in a single-handed medical specialist practice led by one of the authors, AB, an orthopaedic shoulder specialist and DEGUM (German Society for Ultrasonography in Medicine) certified instructor in ultrasonographic shoulder diagnosis. The physiotherapy treatment took place in 24 collaborating physical therapy practices in the broader area of Hamburg. (In our protocol, we initially considered seven collaborating practices, but expanded their number eventually to 24 to improve recruitment). Recruitment took place between December 2012 to September 2014. Follow-up ended in January 2015.

### Participants

Eligible patients were adults (≥ 18 years) presenting with shoulder pain unrelated to a traumatic event (e.g. an accident) and an ultrasonographically determined PTT who had accepted advice to undergo conservative treatment with physiotherapy (see Table 1 for the full eligibility criteria). These patients typically present with clinical signs of “shoulder impingement”, such as a painful arc or positive “impingement signs” [7, 9, 10]. We additionally determined the presence of a PTT by diagnostic ultrasonography, which is highly specific for detecting PTTs [31]. Our intention was to recruit patients whose shoulder pain could reasonably be linked to the presence of a PTT; however, we acknowledge that the precise link between shoulder pain and the presence of a PTT (similar to other shoulder structures) is unclear [32]. Following standard practice, the assessment involved a structured patient history, physical and ultrasonographic evaluation. The physical evaluation was based on DVSE (German Society for Shoulder and Elbow Surgery) recommendations [33]. The ultrasonographic evaluation followed DEGUM and DGOU (German Society for Orthopaedics and Trauma) standards [34]. An ultrasound unit within the highest DEGUM appliance class was used together with a linear transducer with a resolution of ≥10 MHZ and width of ≥40 mm. Diagnosis of a rotator cuff defect was based on alterations of structure and form, following the criteria of Hedtmann & Fett [35, 36]. In distinction to a PTT, a full-thickness tear (FTT) was marked by the absence of a depiction of the rotator cuff (discontinuity of the cuff).

**Table 1:** Eligibility criteria

Inclusion: ▪ Patients with (local) shoulder pain in the presence of an atraumatic (ultrasonographically detected) partial thickness tear ▪ Clinical signs of ‘shoulder impingement’ (e.g. painful arc, positive impingement tests) ▪ Adults (≥ 18 years) ▪ No restrictions on sex ▪ Agreement on conservative treatment ▪ Ability to speak and comprehend the German language ▪ Agreement to participate (signed informed consent) ▪ Anticipated availability for follow-up (living in area of Hamburg) ▪ Agreement to physiotherapy in a collaborating practice Exclusion: ▪ Presence of a full thickness tear at the affected shoulder ▪ Previous substantial shoulder trauma (e.g. shoulder dislocation, fractures) ▪ Previous surgery for the affected shoulder ▪ Previous surgery in the shoulder area that may be causal of or contributory to the current problem (e.g. surgery for breast cancer) ▪ Clinically relevant glenohumeral degeneration or disease (e.g. frozen shoulder) ▪ Current glenohumeral septic arthritis ▪ Clinically relevant acromioclavicular arthritis (e.g. local tenderness, positive provocation tests) ▪ Clinically relevant calcific tendinitis ▪ Ultrasonographic evidence of long head of biceps (LHB) tendon subluxation/ dislocation ▪ Referred pain from the cervical spine region ▪ Multisite musculoskeletal pain ▪ Systemic disorders, diseases or comorbidities as potential sources of (the current) shoulder pain (e.g. breast cancer, rheumatoid disease, inherited disorders (e.g. Marfan syndrome, Ehlers-Danlos syndrome)), or as impairing treatment (e.g. cancer, cardiac insufficiencies) ▪ Neurological disorders or deficits as potential sources of (the current) shoulder pain or impairing assessment and treatment (e.g. hemiplegic shoulder) ▪ Worker’s compensation claims ▪ Unwillingness or inability to give informed consent (e.g. cognitive or intellectual impairments)	

### Treatment

Participants were followed over three months of standard conservative care with physiotherapy in one of the collaborating practices. Adjunctive medical treatment (e.g. local steroid injections), was delivered by AB where considered appropriate. The physiotherapy treatment followed a broad best-evidence protocol based on two systematic reviews [37, 38]. These reviews provided evidence supporting exercises with or without manual therapy as the first-line approach for treating patients with rotator cuff related shoulder pain including PTTs, but could not provide conclusive guidance on the optimal type or dose of treatment. Since there was no justification for restricting treatment to any specific exercises or manual techniques, the protocol was based on the broad principles that a) exercises, preferably combined with manual techniques (soft tissue and/or joint mobilisation), would be the key treatment components, and b) flexibility of the interventions and in the provision of adjunctive modalities would be allowed. In keeping with the ethos of an observational study, the specific content and amount of treatment were unregulated, i.e. individually advised. Treatment, which included the clinical follow-up appointment at three months to assess progress and need for further treatment, was delivered in compliance with German healthcare regulations and AB’s standard practice. Acceptability of the physiotherapy protocol was confirmed by all collaborating physiotherapy practices. Treatment details were documented in a purpose-designed, piloted report form.

### Outcomes

The primary outcome, the outcome to be predicted, was the change in ‘disability’ (disability and health-related quality of life) from baseline to follow-up, measured by a validated German version of the Western Ontario Rotator Cuff Index (WORC) [39, 40]: WORC_CHANGE_. The WORC has been shown to be a valid, reliable and responsive patient-reported outcome measure (PROM) for use in people with rotator cuff disorders [41, 42]. It comprises 21 questions. Responses are made by putting a mark on a 100 mm visual analogue scale (VAS), with lower scores indicating less disability. Scores range from 0 to 2100 [39]. We adjusted all WORC_CHANGE_ values for Regression to the Mean (RTM) using methods outlined by Linden [43]. Participants completed questionnaires at baseline and at 3 to 4 months, the study endpoint, either at AB’s clinic or at home.

As both the WORC and all prognostic factors were patient-assessed, there was no blinding of participants. Nonetheless, the WORC was completed independently and in the absence of AB and study investigators.

Secondary outcomes were tear progression, defined as the presence (yes or no) of an FTT at follow-up, and participants’ perceived overall change of their shoulder problem, measured by a 7-point Global Perceived Change (GPC) scale (from − 3 = “worse as ever” to + 3 = “completely recovered”). Lastly, physical therapy-related adverse events were monitored.

### Prognostic factors

**Fig. 1:**
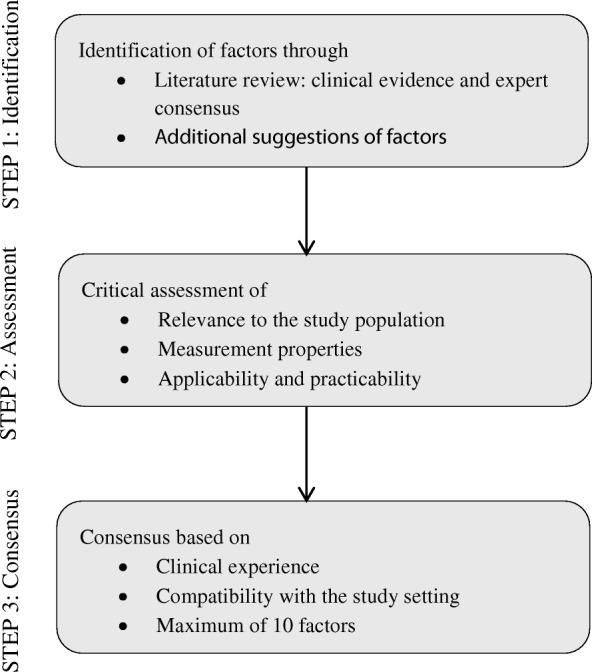
Identification and selection of candidate factors – outline of process

Inclusion of candidate factors was restricted to factors from the baseline assessment, regardless of their type (e.g. demographic, physical). Selection was done through a systematic, three-stage approach comprising identification of factors, critical assessment of these, and a consensus phase that aimed to select a maximum of 10 factors (see Fig. 1 for an outline of the process; a full account is available in Braun 2016 ([29, Chapter 5]). The process was informed by comprehensive literature searches of several electronic databases, including Medline, Embase and Cinahl, for primary prognostic studies, prognostic systematic reviews and expert consensus studies. We screened overall around 3900 records and identified 23 primary study reports (relating to 22 studies), one systematic review and one expert consensus study as relevant sources for informing the selection of factors for our study (a list of these articles is provided in Additional file 1). We extracted and considered 36 factors altogether (these are listed in Additional file 2, which also shows for each factor whether it was included or excluded and the reasons for exclusion). We assessed the relevance of all factors to the study population and setting, their measurement properties, practicality of use, and their applicability, and excluded those that were either not relevant to the study population and setting, not sufficiently valid and reliable, or not applicable in most clinical settings. We grouped the remaining factors according to the availability of clinical evidence and expert consensus supporting their prognostic relevance; we gave preference to the selection of those factors for which there was reasonably consistent support for their prognostic relevance, either through clinical evidence from several studies, or from both clinical evidence and expert consensus. Notably, there was reasonably consistent evidence of prognostic value from several studies pertaining to clinical outcomes of conservative treatment in adults with rotator cuff disorders for only three factors: age, disability and symptom duration. We finally agreed on 10 factors: age, sex, physical demands, disability, pain, history of shoulder pain, symptom duration, diabetes, smoking and pain catastrophizing. We gave thorough attention to factor definitions and measurements (Table 2). All factors were assessed during the patients’ baseline appointment with AB. Since the study was prospective, the assessment of prognostic factor information was inherently blinded to knowledge about the outcome.

**Table 2:** Candidate factors – definition and measurement

No	Predictor variable	Measure / measurement system
1	Age	Age at initial presentation (years)
2	Sex	Sex (female, male)
3	Physical demands	”Before you had your current shoulder problem, did a typical week include one or more of the following activities (yes, no): ▪ Repetitive or prolonged use of the affected arm for strength effort (e.g. lifting, carrying or moving heavy loads, athletic sports, strength-demanding skilled manual work) ▪ Repetitive or prolonged use of the arm above shoulder height (e.g. overhead work, overhead sports, throwing sports, work as a hairdresser)?”
4	Disability	Western Ontario Rotator Cuff Index (WORC) [39]; validated German version [40] (score)
5	Pain	“What is the worst amount of pain that you have experienced within the past week?” (100 mm visual analogue scale VAS)
6	History of shoulder pain (incl. Previous treatment)	“Prior to the current episode, have you ever seen a medical doctor or therapist for pain in this shoulder?” (yes, no)
7	Symptom duration	“For how long have you been having your current shoulder complaints?” (weeks)
8	Diabetes	“Do you have diabetes?” (yes, no)
9	Smoking	“Are you a smoker? Please tick “yes” if you regularly smoke at least once a week any amount of tobacco” (yes, no)
10	Pain catastrophizing	Pain Catastrophizing Scale (PCS) [56]; validated German version [57] (score)

### Sample size

The multivariable nature of prognostic model studies makes it difficult to estimate the required sample size [26]. Indeed, no formal methods (based on either power calculations or adequate precision of estimation of effects) are available to determine the effective sample size, and recommendations for the sample size vary across the literature. Following work by Vittinghoff & McCulloch [44], we based the minimum sample size of our study on a requirement of 5 to 9 outcome events (events equate to individuals for continuous outcomes) per candidate prognostic factor in relation to the full model (i.e. the model including all 10 factors). As per our protocol, we initially planned to analyze the WORC as a binary outcome variable, but subsequently (and prior to the analysis) decided to analyze it as a continuous variable to avoid the unnecessary loss of information that would have resulted from dichotomization [45, 46]. By analyzing the WORC on a continuous scale, and setting out to study overall 10 factors, which we considered feasible, we aimed to include (5 to 9)*10 = 50 to 90 participants. Increased by 20% to allow for losses to follow-up, the recruitment target was 60 to 108 patients.

### Missing data

Any missing prognostic factor and outcome data were documented. The decision about the method for dealing with missing data, including whether or not to impute any missing data, was made prior to the analysis. We considered the amount and also the potential reasons for missing values, i.e. whether the reasons for missingness appeared systematic or random. We decided to limit the replacement of missing values to those missing for the two multi-item measures, the WORC (baseline and follow-up) and the Pain Catastrophizing Scale (PCS). No standard missing rule was available for the WORC in the literature; therefore, we replaced missing WORC values by the mean of the respective domain. We replaced missing PCS values by the mean of the items that were completed, as suggested by the primary originator of the scale, Prof Michael Sullivan (personal communication 02/06/2014). We did not replace any missing values where the PCS was completely missing. As the information-theoretic analysis approach we used required identical datasets, the data were analyzed on a complete-case basis. We would have considered formal testing of the effects of missing data should the amount have been bigger and should the reasons for missingness have been of concern.

### Statistical analysis methods

We intended to include all 10 candidate factors in the prognostic modelling analysis. All continuous factors, WORC and PCS scores, were analyzed as continuous measurements. All non-continuous factors were binary.

We based our analysis on an information-theoretic approach, namely on a small-sample variant of Akaike’s Information Criterion (AIC) approach, AIC_C_ [47]. Information-theoretic approaches to model selection differ from other approaches, particularly from the widely used stepwise regression approaches, in several ways. Under the AIC approach, selection is based on the comparison of multiple candidate models, which are pre-specified based on “theory”, rather than on a single global set of factors [48]. Selection is further based on an information-theoretic criterion (e.g. AIC), which provides “numerical values that represent the scientific evidence” for a model, but no “test statistics” such as *p* values, thus avoiding the application of arbitrary cut-offs of “statistical significance” ([47] p. 64). Reflecting the perspective that models never reflect “full reality”, i.e. that they are approximations ([47], p. 27), the AIC value represents an estimator of the information that is inherently lost when a model is used to approximate full reality (Kullback-Leibler information) [48]. The AIC accounts for the number of candidate factors by ‘penalizing’ models with larger numbers of factors, thereby favouring parsimony ([47], p. 60–1). The model with the lowest AIC value (AIC_MIN_) represents the closest approximation and is accordingly termed the “best model” within a set of models [47]. AIC differences (∆AIC = AIC – AIC_MIN_) can then be calculated to rank the models by their distance to the best model [47, 48]. Burnham et al. ([48], p. 25) have proposed considering models with ∆AIC values < 4 to 7 as “plausible” alternatives to the best model, whereas models with higher ∆AIC values (> 9) have little to no support. AIC values are relative rather than absolute, and “on the scale of information” ([47], p. 84). Accordingly, their use is limited to comparing models within a defined set of models [49]. As the AIC approach will always select a best model among a set of models, it has been suggested that the worth of the best or the global (full) model be assessed, e.g. by a goodness-of-fit test, analysis of residuals or the adjusted R^2^ (the percentage of variance explained) [47].

Following recommendations from the literature that the number of candidate models should usually be limited to a few [47], we decided to analyze a selection of nine candidate models. The selection of models was based on clinical and theoretical considerations, with the first model (number 1 in Table 3) including all 10 candidate prognostic factors (thus representing the “full model”). The composition of the other eight models, which included between two to eight of these factors, was based on various characteristics, as shown in Table 3. Examples of characteristics were the potential for modification (model 2) or the effort required for the assessment of prognostic factors (models 5 and 7, inclusion or exclusion of questionnaires), which would be highly relevant to clinical practitioners. The primary analysis approach was a linear regression analysis [26, 49] which we conducted in IBM SPSS Statistics 22. All continuous factors were modelled as linear. Satisfaction of the assumptions of linear regression was assessed visually for each model based on the residual plot (scatterplot of standardized residuals against standardized predicted values) [50].

**Table 3:** Candidate prognostic models and key model statistics

No	Candidate model	N^*^ factors	Main characteristic	AIC_C_	∆AIC_C_^†^	SEE	R^2^_ADJ_^§^
1	Age + sex + physical demands + disability (WORC) + pain + history of shoulder pain + symptom duration + smoking + pain catastrophizing (PCS) (+ diabetes removed^‡^)	9	Full model (all factors)	891	11	313	0.12
2	Smoking + pain catastrophizing (PCS) (+ diabetes removed^‡^**)**	2	Potential for modification (could be modified (addressed) by some action (e.g. treatment)	880	0	314	0.11
3	Age + sex	2	Factors that cannot be modified	889	9	336	−0.02
4	Age + sex + physical demands + pain + history of shoulder pain + symptom duration + smoking (+ diabetes removed^‡^)	7	Type of assessment: “no questionnaires”	899	19	344	−0.06
5	Disability (WORC) + pain catastrophizing (PCS)	2	Type of assessment: “questionnaires”	880	0	314	0.11
6	Smoking (+ diabetes removed^‡^)	(1)	Type of factor: “bio(logical) factors”	Excluded from analysis due to removal of diabetes			
7	History of shoulder pain + symptom duration	2	Background (patient history)	889	9	336	−0.02
8	Pain + history of shoulder pain + symptom duration	3	Further models: pain-related factors (excluding pain catastrophizing)	889	9	335	−0.01
9	Pain + pain catastrophizing (PCS)	2	Further models: pain and attitude towards pain	882	2	318	0.09

^*^Denotes the number of factors in each model as analyzed (i.e. after removal of diabetes). ^**†**^An ∆AIC_C_ value of 0 denotes the model(s) with the lowest AIC_C_ value(s), representing the “best” model(s) within the set of candidate models; ^‡^Model initially included diabetes, which was excluded from the analyses due to its low prevalence in the sample; ^§^Negative R^2^ values are generally interpreted as “0”

We extracted the following statistics: the AIC_C_ value; the standard error of the estimate (SEE), as the primary measure of model precision; the adjusted coefficient of (multiple) determination (R^2^_ADJ_), as a complementary measure of model performance; the regression constant (Constant); and the unstandardized regression coefficients (B) of all factors with their 95% confidence intervals (CIs). For comparison of the different models, we extracted AIC_C_, ∆AIC and SEE values.

### Model validation and further analyses

We intended to compare the SEE of the best model with the estimate of the Minimal Important Difference (MID) of the WORC, which we intended to derive from the sample data, and to internally validate any model with an SEE substantially lower than the MID. We intended to conduct the following exploratory subgroup analyses: amount of physiotherapy (number of sessions); medical treatment (specifically provision of injections); and length of follow-up.

## Results

### Participants

**Fig. 2:**
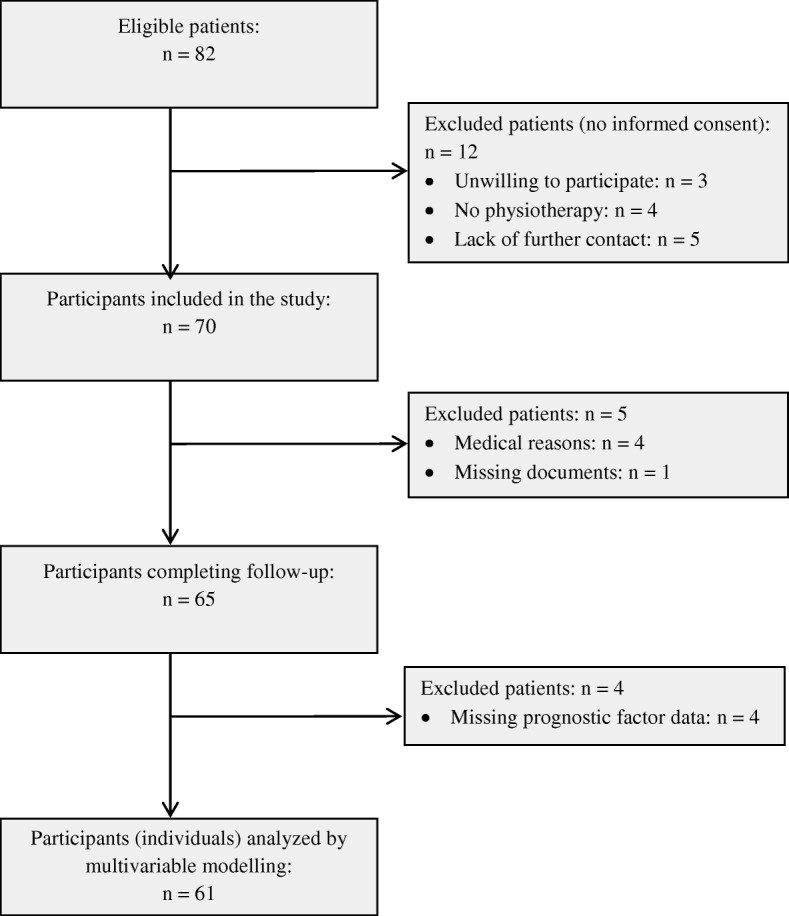
Flow of participants

Figure 2 illustrates the flow of participants. Of 82 eligible participants, 70 were included, of whom 65 (representing 65 shoulders) completed the study. The baseline characteristics and prognostic factor information of these 65 participants are presented in Table 4.

**Table 4:** Baseline characteristics and prognostic factor data

Characteristic (*n*)	Measurement	Values		
Continuous prognostic factors			SD	Range
Age (65)	year	50	12	24–76
Disability (65)*	WORC_1 score	897	380	130–1660
Pain (64)	mm VAS	63	26	7–100
Symptom duration (63)	week	36	49	1–250
Pain catastrophizing (62)*^†^	PCS score	15	9	1–37
Categorical prognostic factors		N	%	
Sex (65)	female	25	38	
	male	40	62	
Physical demands (64)	yes	41	64	
	no	23	36	
History of shoulder pain (64)	yes	35	55	
	no	29	45	
Diabetes (65)	yes	4	6	
	no	61	94	
Smoking (64)	yes	10	16	
	no	54	84	
Additional characteristics		N	%	
Affected tendon (65)	1. supraspinatus	63	97	
	2. infraspinatus	1	2	
	3. supraspinatus + infraspinatus	1	2	
	4. any other	0	0	
Dominant arm affected (65)	yes	46	71	
	no	19	29	
Work status (64)	5. full-time	41	64	
	6. part-time	11	17	
	7. sick leave	0	0	
	8. retired	10	16	
	9. not working (other reason)	2	3	

^*^Includes replaced values for missing data (see section 6.6.15); ^**†**^PCS data were completely missing for three cases

The amount of missing data was small: six values (0.4% of all values) were missing for the baseline WORC; 11 (1%) for the follow-up WORC; and six (1%) for the single-item prognostic factors. The PCS was missing completely for three participants; beyond this, only one PCS value (0.1%) was missing. The distribution appeared random, thus non-systematic. Four participants had missing prognostic factor data after replacement of missing WORC and PCS values, and were consequently, in keeping with the need for identical datasets for the AIC approach [47], excluded from the modelling. The data of 61 participants were analyzed. The mean (SD) interval between completion of the baseline and follow-up WORC (and GPC) was 97 (17) days (*n* = 65 for WORC, 64 for GPC). The mean (SD) interval between the baseline and follow-up US assessment was 100 (13) days (*n* = 52).

### Treatment

All participants received conservative treatment with physiotherapy. The mean (SD) number of physiotherapy sessions was 12 (6); and the mean (SD) duration of single sessions was 28 (13) minutes. A breakdown of the physiotherapy treatment content, documented by the physiotherapists, is provided in Table 5. Treatment usually included a combination of exercises and manual techniques. Consistent with physiotherapy practice in Germany, where this study took place, all physiotherapists routinely provided advice and patient education.

**Table 5:** Breakdown of physiotherapy treatment

Category	Domain (*n* = 65)	*N*	%
Types of exercises	Strengthening exercises focused at rotator cuff muscles	52	80
	Scapula positioning exercises	47	72
	Stabilisation exercises	41	63
	Stretching techniques or exercises (shoulder/shoulder girdle)	36	55
	Strengthening exercises focused at shoulder girdle muscles	34	52
	Humeral head ‘positioning’ exercises	33	51
	Coordination exercises	25	38
	Inclusion of high load exercises (> 80% RPM^†^)	5	8
	Correction of thoracic spine posture^*^	2	3
	Proprioceptive Neuromuscular Facilitation (PNF)^*^	1	2
Types of exercise equipment	Use of small equipment (e.g. elastic bands)	45	69
	Use of training machines (e.g. pulley, pull-down)	27	42
Setting of exercise treatment	Provision and supervision of supplementary home exercises	42	65
Types of manual techniques	Soft tissue techniques (shoulder or shoulder girdle)	56	86
	Manual mobilisation techniques (shoulder)	51	78
	Manual mobilisation of thoracic spine^*^	9	14
	Manual mobilisation of ribs^*^	2	3
	Manual mobilisation of cervical spine^*^	2	3
Supplementary modalities	Heat or cold applications	14	22
	Therapeutic ultrasound^*^	1	2

Interventions are listed by general category and specific domain; domains are in descending order of use; ^*^recorded in “anything else?” category (physiotherapy report form); ^**†**^RPM = one-repetition maximum

Thirty-seven participants (57% of 65) received some supplementary medical treatment: i.e. subacromial steroid injection (27; of these, 24 received one injection and three received two injections), elastic tape (12) or prescription of oral medication (Metamizole, 1). No participant was put on sick leave.

### Outcomes

The mean (SD) unadjusted WORC_CHANGE_ score (*n* = 65) was − 363 (361); the range was − 1248 to 372. The mean (SD) RTM-adjusted WORC_CHANGE_ score was − 363 (341); the range was − 1102 to 387. Tear progression to an FTT occurred in two participants (4%, *n* = 52). Adverse events were reported for six participants (9%, *n* = 65), and related exclusively to temporary exacerbations of the shoulder symptoms. Fifty-five participants (86%, *n* = 64) rated their shoulder problem as improved (positive GPC ratings), five (8%) as unchanged (GPC = 0), and four (6%) as deteriorated (negative GPC ratings). The MID estimate for the WORC, which we derived from the sample data using an anchor-based approach (*n* = 64), was − 300 (this analysis is reported in a separate article [51]).

### Prognostic modelling

There were no complexities (e.g. unit of analysis issues) in the data. We excluded diabetes from the analysis because of its very low prevalence in the sample (Table 4), and consequently excluded one two-factor model, ‘diabetes & smoking’ (Table 3). The ratio of the number of outcome events (individuals with data available for analysis) to the overall number of analyzed candidate factors approximated to 7 (61/9); the range across all models was, depending on the number of factors included in each model, approximately 7 to 31. The residual plots showed no strong evidence of a violation of the assumptions for linear regression for any of the models.

The key model statistics are shown in Table 2. The coefficient statistics for each model and each prognostic factor are provided with the supplementary materials (Additional file 3). Two models with the same AIC_C_ value (models 2 and 5) were identified as the best models. The model with the third-highest AIC_C_ value (model 9) had an ∆AIC_C_ within the range of plausible alternatives (∆AIC_C_ < 7) to the best models [48]. The remaining models had ∆AIC_C_ values outside this range. The SEE ranged from 313 to 344, and was, for all models, higher than the estimated MID of the WORC (300). The full model (model 1) had the highest R^2^_ADJ_ (the range of all models was from − 0.06 to 0.12).

### Model validation and further analyses

The performance and precision of the analyzed models did not justify internal validation; nor the planned subgroup analyses.

## Discussion

### Principal findings

Despite our rigorous approach and meeting our minimum sample size (relating to the full model), we did not achieve our primary aim of developing a prognostic model for the outcome of a phase of conservative treatment with physiotherapy in adults with symptomatic atraumatic rotator cuff PTTs. Of the eight models for which testing was appropriate, none had a satisfactory performance (R^2^_ADJ_) or precision (SEE).

### Strengths and weaknesses of the study

The rigorous methodological design of our study helped to avoid various potential sources of bias. This included avoidance of statistical univariable selection techniques, which have been linked to biased predictions [52], and the analysis of continuous measurements on their continuous scale, hereby avoiding the various problems associated with the categorization of continuous measurements [45, 46]. The latter reflected our post-protocol decision to analyze the WORC on a continuous scale, instead of analyzing it as a binary outcome. By using an information-theoretic analysis approach, we purposely avoided the selection of factors within the multivariable analysis based on arbitrary cut-offs of “statistical significance”, as these, in particular stepwise regression techniques, have been linked to biased predictions [52–54]. Although the outcome assessment could not be blinded to the prognostic factor information, any influence of participants’ knowledge about prognostic factor information on the outcome is unlikely because the participants did not know which of the multiple baseline variables were modelled.

The ratio of outcome events to candidate factors was within the pre-specified range of 5 to 9 for the full model (and considerably higher, i.e. > 20, for some of the other models), and losses to follow-up and missing data were few. Additionally, as the reasons for missingness appeared non-systematic, we considered the data from the complete cases as representative of the whole sample. However, despite our meeting our sample size estimate, sample size is a key limitation of our study as indicated by the low precision and also by the rejection of the ‘diabetes & smoking’ model due to the low numbers of diabetic patients recruited. In the absence of any formal methods to determine the effective sample size, and without prior knowledge of the relationship between the candidate prognostic factors, it was difficult to estimate the sample size for our study (please see reviewer feedback on this aspect in Open Peer Review Reports). Considering the low precision of the analyzed models in our study, we conclude that a much larger sample size would have been needed to increase the chances of achieving satisfactory precision of the analyzed models.

Rigour was applied to the consideration of the clinical relevance, practicality of measurement and applicability of the study findings. All PTTs were diagnosed by US, which is highly specific (94%), but less sensitive (68%) for detecting PTTs [31]. This means that, while some PTTs might have been missed, those identified were almost certainly true positives; hence, the study population was homogeneous in this respect. We aimed to enroll patients at a fairly similar state of health. Similarity of several baseline characteristics such as pain intensity, symptom duration and disability could not be guaranteed, as their restriction would have threatened recruitment, but was accounted for by candidate prognostic factors.

The physiotherapy protocol accommodated clinical autonomy within an evidence-based framework. Some of the study participants received adjunctive medical treatment, such as a local steroid injection. Arguably, the different treatments may have had an impact on the overall improvement of the participants during the three- month treatment period and also on the predictive performance of the analyzed models. We are confident, though, that this was not a relevant issue in our study. Consistent with our study question, we selected prognostic factors that were present at baseline before starting conservative treatment. The primary treatment was exercise-based physiotherapy within an evidence-based framework. The adjunctive treatments, which were provided to a minority of participants, included subacromial corticosteroid injections, elastic tapes and oral pain medication. The evidence on the effectiveness of these treatments for rotator cuff related shoulder pain is limited. Notably, for corticosteroid injection, which was the most often delivered adjunctive treatment, there is evidence of no relevant difference compared with physiotherapy [55]. Considering this and that the majority of the participants in our study who received injections received only one injection, we consider the likely impact of corticosteroid injections was minimal. Similar considerations apply to the other adjunctive treatments, which were received by smaller numbers of participants. In this context, we consider our decision not to perform the planned exploratory subgroup analyses, which included “medical treatment (specifically provision of injections)”, was appropriate.

Although set within one country, Germany, with clinical care under one orthopaedic specialist, the study findings are broadly applicable to adults with symptomatic PTTs undergoing a three-month period of conservative treatment with exercise-based physiotherapy.

The eight analyzed models could explain only a very limited amount (up to 12%, see R^2^_ADJ_ values), of the variability of the outcome, which means that most of the variability remains unexplained. This finding could be partly due to the fact that the evidence base for most of the factors identified was generally very limited. Although we cannot say what other factors may have contributed to this unexplained variability, we suggest these may be among the 36 factors listed in the supplementary table. As evidenced by their low precision (SEE), the predictions are affected by considerable uncertainty; they consequently do not provide reliable estimates of population parameters. The “natural” temptation to select out more “promising” factors, such as pain catastrophizing, which featured in the three best models, should be countered by the realization that our study was explicitly designed to explore multivariable models rather than individual factors. Thus, the presented coefficient statistics do not represent the factors’ independent contributions to the predictions.

Lastly, it should be kept in mind that generally, any prognostic model that has been developed in a single population should only be considered clinically usable after it has been externally validated and, ideally, also evaluated for clinical impact [25].

### Comparison with other studies

As already established, this is the first study aimed at predicting the outcome of conservative treatment with physical therapy in adults with symptomatic PTTs. Comparison with studies of adults undergoing conservative treatment with physiotherapy for rotator cuff disorders, in general, would be uninformative because of heterogeneity, not least in methodological terms [28].

## Conclusions

We could not determine a prognostic model with satisfactory performance and precision. Thus, the challenge remains to develop a prognostic model with a satisfactory performance and precision for predicting the outcome of a phase of conservative treatment with physiotherapy in adults with symptomatic PTTs. Further high-quality prognostic studies are needed but should be underpinned, and thus preceded, by robust research aimed at improving knowledge of relevant factors. Consensus approaches (e.g. Delphi studies) may provide guidance about which factors to prioritize for future studies. Collaborative data collection and data sharing initiatives could enhance the realization of larger studies and applicability. Further methodological research is also needed to determine the optimal methods for developing prognostic models. Investigators of future prognostic model development studies should attend to the importance of the internal and external validation of any models with a promising performance.

## Additional files

Additional file 1: Primary study reports and other articles used to identify prognostic factors (DOCX 21 kb)

Additional file 2: Factors considered for inclusion in the study (DOCX 20 kb)

Additional file 3: Model coefficient statistics (DOCX 21 kb)

## Abbreviations

**∆**AIC_C_: AIC_C_ difference
ADJ**,**_ADJ_: Adjusted (in the context of this study: for regression to the mean, RTM)
AIC: Akaike’s Information Criterion
AIC_C_: Akaike’s Information Criterion, small sample variant
AIC_MIN_: Smallest AIC (AIC_c_) value
CI: Confidence interval
DEGUM: Deutsche Gesellschaft für Ultraschall in der Medizin [German Society for Ultrasound in Medicine]
DVSE: Deutsche Vereinigung für Schulter- und Ellenbogenchirurgie [German Society of Shoulder and Elbow Surgery]
FTT: Full-thickness tear (rotator cuff)
GPC: Global Perceived Change
LHB: Long head of biceps
MID: Minimal Important Difference
MRI: Magnetic resonance imaging
PCS: Pain Catastrophizing Scale
PROM: Patient-reported outcome measure
PTT: Partial-thickness tear (rotator cuff)
R^2^: Coefficient of (multiple) determination
RTM: Regression to the mean
SD: Standard deviation
SEE: Standard error of the estimate
TRIPOD: Transparent Reporting of a Multivariable Prediction Model for Individual Prognosis or Diagnosis (checklist)
US: Ultrasonography
VAS: Visual analogue scale
WORC: Western Ontario Rotator Cuff index
WORC_1: Baseline WORC
WORC_2: Follow-up WORC
WORC_change: Change of WORC score from baseline to follow-up
