# Supplementary material for: Predicting the outcome of conservative treatment with physiotherapy in adults with shoulder pain associated with partial-thickness rotator cuff tears – a prognostic model development study
Source: BMC Musculoskelet Disord. 2018 Sep 11;19:329. doi: 10.1186/s12891-018-2239-8 (PMC6134519; doi:10.1186/s12891-018-2239-8)
Supplement: Supplementary file 1 — Primary study reports and other articles used to identify prognostic factors (DOCX 21 kb) [file 12891_2018_2239_MOESM1_ESM.docx]

# Additional File 1: Primary study reports and other articles used to identify prognostic factors.

Articles are listed by type - primary (clinical) study, systematic review, expert consensus.

| **No** | **Study ID*** | **Reference** |
| --- | --- | --- |
| ***Primary study*** | | |
| 1 | Bartolozzi 1994 | Bartolozzi A, Andreychik D, Ahmad S. Determinants of outcome in the treatment of rotator cuff disease. Clin Orthop Relat Res. 1994;308:90-7. |
| 2 | Chard 1988 | Chard MD, Sattelle LM, Hazleman BL. The long-term outcome of rotator cuff tendinitis--a review study. Br J Rheumatol. 1988;27: 385-9. |
| 3 | Conroy 1998 | Conroy DE, Hayes KW. The effect of joint mobilization as a component of comprehensive treatment for primary shoulder impingement syndrome. J Orthop Sports Phys Ther. 1998;28:3-14. |
| 4 | Cummins 2009 | Cummins CA, Sasso LM, Nicholson D. Impingement syndrome: Temporal outcomes of nonoperative treatment. J Shoulder Elbow Surg. 2009;18:172-7. |
| 5 | Ekeberg 2010 | Ekeberg OM, Bautz-Holter E, Juel NG, Engebretsen K, Kvalheim S, Brox JI. Clinical, socio-demographic and radiological predictors of short-term outcome in rotator cuff disease. BMC Musculoskelet Disord. 2010;11:239. |
| 6 | Engebretsen 2010 | Engebretsen K, Grotle M, Bautz-Holter E, Ekeberg OM, Brox JI. Predictors of shoulder pain and disability index (SPADI) and work status after 1 year in patients with subacromial shoulder pain. BMC Musculoskelet Disord. 2010;11:218. |
| 7 | Hardy 1986 | Hardy DC, Vogler JB, White RH. The shoulder impingement syndrome: prevalence of radiographic findings and correlation with response to therapy. *AJR Am J Roentgenol. 1986;* 147(3), 557-61. |
| 8 | Hawkins 1995 | Hawkins R. H., Dunlop R. Nonoperative treatment of rotator cuff tears. Clin Orthop Relat Res. 1995;321:178-88. |
| 9 | Hung 2010 | Hung C-J, Jan M-H, Lin Y-F, Wang T-Q, Lin J-J (2010). Scapular kinematics and impairment features for classifying patients with subacromial impingement syndrome. Man Ther. 2010;15:547-51. |
| 10 | Itoi 1992 | Itoi E, Tabata S. Conservative treatment of rotator cuff tears. Clin Orthop Relat Res. 1992;275:165-73. |
| 11 | Kennedy 2006a | Kennedy CA, Haines T, Beaton DE. Eight predictive factors associated with response patterns during physiotherapy for soft tissue shoulder disorders were identified. J Clin Epidemiol. 2006; 59:485–96. |
| 12 | Kennedy 2006b | Kennedy CA, Manno M, Hogg-Johnson S, Haines T, Hurley L, McKenzie D, Beaton DE. Prognosis in soft tissue disorders of the shoulder: predicting both change in disability and level of disability after treatment. Phys Ther. 2006;*86*:1013–32; discussion 1033–7. |
| 13 | Maman 2009 | Maman E, Harris C, White L, Tomlinson G, Shashank M, Boynton E. Outcome of nonoperative treatment of symptomatic rotator cuff tears monitored by magnetic resonance imaging. J Bone Joint Surg Am. 2009;91:1898-906. |
| 14 | Morrison 1997 | Morrison DS, Frogameni AD, Woodworth P (1997). Non-operative treatment of subacromial impingement syndrome. J Bone Joint Surg Am. 1997;79:732-7. |
| 15 | Safran 2011 | Safran O, Schroeder J, Bloom R, Weil Y, Milgrom C. Natural history of nonoperatively treated symptomatic rotator cuff tears in patients 60 years old or younger. Am J Sports Med. 2011;39:710-4. |
| 16 | Selvanetti 1998 | Selvanetti A, Giombini A, Caruso I. Nonoperative treatment of partial-thickness rotator cuff tears in overhead athletes. Med Sci Sports Exerc. 1998;30:S260. |
| 17 | Taheriazam 2005 | Taheriazam A, Sadatsafavi M, Moayyeri A. Outcome predictors in nonoperative management of newly diagnosed subacromial impingement syndrome: a longitudinal study. MedGenMed. 2005;7:63. |
| 18 | Tanaka 2010 | Tanaka M, Itoi E, Sato K, Hamada J, Hitachi S, Tojo Y, Honda M, Tabata S. Factors related to successful outcome of conservative treatment for rotator cuff tears. Ups J Med Sci. 2010; 115:193-200. |
| 19 | Vad 2002 | Vad VB, Warren RF, Altchek DW, O’Brien SJ, Rose HA, Wickiewicz TL. Negative prognostic factors in managing massive rotator cuff tears. Clin J Sport Med*.* 2002;12:151-7. |
| 20 | Virta 2009 | Virta L, Mortensen M, Eriksson R, Möller M. How many patients with subacromial impingement syndrome recover with physiotherapy? A follow-up study of a supervised exercise programme. Adv Physiother. 2009;11:166-73. |
| 21 | Wang 2000 | Wang JC, Horner G, Brown ED, Shapiro MS. The relationship between acromial morphology and conservative treatment of patients with impingement syndrome. Orthopedics. 2000;23:557–9. |
| 22 | Wu 2003 | Wu HP, Dubinsky TJ, Richardson ML. Association of shoulder sonographic findings with subsequent surgical treatment for rotator cuff injury. J Ultrasound Med. 2003;22:155-61. |
| 23 | Yamanaka 1994 | Yamanaka K, Matsumoto T. The joint side tear of the rotator cuff. A followup study by arthrography. Clin Orthop Relat Res. 1994;304:68–73. |
| ***Systematic review*** | | |
| 24 | Kuijpers 2004 | Kuijpers T, van der Windt DAWM, van der Heijden GJMG, Bouter LM. Systematic review of prognostic cohort studies on shoulder disorders. Pain. 2004;109:420-31. |
| ***Expert consensus*** | | |
| 25 | Vergouw 2011 | Vergouw D, Heymans MW, de Vet HC, van der Windt DA, van der Horst HE. Prediction of persistent shoulder pain in general practice: Comparing clinical consensus from a Delphi procedure with a statistical scoring system. BMC Fam Pract. 2011;12:63. |

* First author, year
