# Supplementary material for: Predicting the outcome of conservative treatment with physiotherapy in adults with shoulder pain associated with partial-thickness rotator cuff tears – a prognostic model development study
Source: BMC Musculoskelet Disord. 2018 Sep 11;19:329. doi: 10.1186/s12891-018-2239-8 (PMC6134519; doi:10.1186/s12891-018-2239-8)
Supplement: Supplementary file 2 — Factors considered for inclusion in the study (DOCX 20 kb) [file 12891_2018_2239_MOESM2_ESM.docx]

**Additional File 2:** **Factors considered for inclusion in the study, with reasons for exclusion**

A full account of the process for identifying and selecting the candidate factors for the study is available in Braun 2016 (Chapter 5): <http://tees.openrepository.com/tees/handle/10149/621790>.

| **No** | **Factor** | **Included (INCL) or excluded (EXCL) 🡪 reasons for exclusion** |
| --- | --- | --- |
| ***Demographic factors*** | |  |
| 1 | **Age** | **INCL** |
| 2 | Education | EXCL at final consensus stage 🡪 other factors were prioritized |
| 3 | **Sex** | **INCL** |
| ***Activity-related factors*** | |  |
| 4 | Dominant arm (affected) | EXCL at final consensus stage 🡪 other factors were prioritized |
| 5 | **Patient’s physical demands** (overhead athletes) | **INCL** |
| ***Symptom-related factors*** | |  |
| 6 | Coexisting neck pain | EXCL at final consensus stage 🡪 other factors were prioritized |
| 7 | **Disability (shoulder-related)** | **INCL** |
| 8 | **Pain** | **INCL** |
| 9 | Patient’s global rating of (severity of) problem | EXCL at final consensus stage 🡪 other factors were prioritized |
| 10 | Quality of sleep (sleep loss due to shoulder pain) | EXCL at final consensus stage 🡪 other factors were prioritized |
| ***Factors related to history of symptoms/shoulder pain*** | |  |
| 11 | Aetiology of symptoms (including history of trauma to the shoulder, type of rotator cuff disease and overuse) | EXCL 🡪 relevance to study: considered as largely irrelevant to my study, as inclusion was restricted to PTTs, i.e. a specific type of rotator cuff disease, and as trauma-related tears were an exclusion criterion. |
| 12 | **History of (previous) shoulder pain** | **INCL** |
| 13 | **Symptom duration** | **INCL** |
| ***Factors from physical examination*** | |  |
| 14 | Impingement sign (presence/absence) | EXCL 🡪 relevance to study: this was among the clinical signs as part of the diagnostic criteria. |
| 15 | Muscle strength (serratus anterior; abduction; rotation) | EXCL 🡪 applicability/practicability: valid measurement would have required equipment that was not available to Dr Betthäuser in his practice (and that is normally not available in the standard practice setting). |
| 16 | Range of motion (active: abduction, external rotation; passive) | EXCL 🡪 relevance to study: not considered a relevant issue to the study population. Also, significant restriction of range of movement was an exclusion criterion. |
| 17 | Scapular kinematics: internal rotation | EXCL 🡪 applicability/practicability: valid measurement would have required specialist equipment that was not available to Dr Betthäuser in his practice (and that is normally not available in the standard practice setting). |
| ***Factors related to comorbidities and (self-reported) health status*** | |  |
| 18 | **Diabetes** | **INCL** |
| 19 | Glenohumeral arthritis | EXCL 🡪 relevance to study: glenohumeral arthritis was an exclusion criterion. |
| 20 | Health status (self reported) | EXCL at final consensus stage 🡪 other factors were prioritized |
| 21 | Multisite pain | EXCL 🡪 relevance to study: multisite pain was an exclusion criterion. |
| 22 | **Smoking** | **INCL** |
| ***Psychosocial/psychological factors*** | |  |
| 23 | Fear-avoidance beliefs | EXCL 🡪 measurement properties: non-availability of an appropriate validated German questionnaire. |
| 24 | Illness perceptions | EXCL at final consensus stage 🡪 other factors were prioritized |
| 25 | **Pain catastrophizing** | **INCL** |
| ***Structural factors (shoulder)*** | |  |
| 26 | Acromion type/morphology | EXCL 🡪 applicability/practicability: assessment requires special X-ray images that are not part of standard practice within German statutory healthcare. |
| 27 | Humeral head migration | EXCL 🡪 relevance to study: clinically relevant glenohumeral degeneration or disease was an exclusion criterion. |
| 28 | Osseous abnormalities (not further specified) | EXCL 🡪 relevance to study: clinically relevant glenohumeral degeneration or disease was an exclusion criterion. |
| ***Rotator cuff specific factors*** | |  |
| 29 | Fatty infiltration | EXCL 🡪 relevance to study: relates mainly to (large) FTTs |
| 30 | Muscle atrophy | EXCL 🡪 relevance to study: relates mainly to (large) FTTs |
| 31 | Tear size (extent) | EXCL 🡪 measurement properties: concerns about validity of measurement of tear size by ultrasound |
| 32 | Type of rotator cuff pathology or tear; tendon integrity | EXCL 🡪 relevance to study: inclusion into the study was restricted to patients with PTTs. |
| ***Interventional factors*** | |  |
| 33 | Corticosteroid injections (response to initial injection; previous) | EXCL 🡪 applicability/practicability: response to initial injection requires all participants to have an injection; this does not comply with standard practice within German statutory healthcare; validity and reliability of measurement: asking about previous injections was considered difficult (participants can’t be expected to know what type of injections they have received). |
| 34 | Medication (regular medication; over-the-counter medication) | EXCL 🡪 relevance to study: Dr Betthäuser’s patients (with PTTs) usually do not take any oral pain medication. |
| ***Economical factors*** | |  |
| 35 | Insurance (worker’s compensation) claims | EXCL 🡪 relevance to study: Dr Betthäuser considered this not to be of any relevance for the study population. |
| 36 | Sick leave | EXCL 🡪 relevance to study: the patients presenting to Dr Betthäuser are usually not on sick leave. |
