# Supplementary material for: Predicting the outcome of conservative treatment with physiotherapy in adults with shoulder pain associated with partial-thickness rotator cuff tears – a prognostic model development study
Source: BMC Musculoskelet Disord. 2018 Sep 11;19:329. doi: 10.1186/s12891-018-2239-8 (PMC6134519; doi:10.1186/s12891-018-2239-8)
Supplement: Supplementary file 3 — Model coefficient statistics (DOCX 21 kb) [file 12891_2018_2239_MOESM3_ESM.docx]

**Additional File 3: Model coefficient statistics**

Abbreviations used in the tables: ADJ = adjusted (for regression to the mean); CI = confidence interval; PCS = pain catastrophizing scale; VAS = visual analogue scale; WORC = Western Ontario Rotator Cuff Index. See main body of article for full details including the model summary statistics.

**Model 1**

| **Constant and factors** | **Unstandardized coefficient B** | **95% CI** | |
| --- | --- | --- | --- |
|  |  | **Lower limit** | **Upper limit** |
| Constant | -729 | -1315 | -144 |
| Age (yr) | 5 | -3 | 12 |
| Sex (female or male) | 47 | -130 | 224 |
| Physical demands (yes or no) | 75 | -108 | 258 |
| Disability (WORC_1_ADJ_ score) | -0.5 | -1 | 0.03 |
| Pain (mm VAS) | 2 | -2 | 6 |
| History of shoulder pain (yes or no) | 50 | -123 | 223 |
| Symptom duration (wk) | -0.4 | -2 | 1 |
| Smoking (yes or no) | 194 | -42 | 430 |
| Pain catastrophizing (PCS score) | 20 | 9 | 32 |

Model 2

| **Constant and factors** | **Unstandardized coefficient B** | **95% CI** | |
| --- | --- | --- | --- |
|  |  | **Lower limit** | **Upper limit** |
| Constant | -599 | -766 | -433 |
| Smoking (yes, no) | 132 | -91 | 355 |
| Pain catastrophizing (PCS score) | 14 | 5 | 23 |

Model 3

| **Constant and factors** | **Unstandardized coefficient B** | **95% CI** | |
| --- | --- | --- | --- |
|  |  | **Lower limit** | **Upper limit** |
| Constant | -542 | -908 | -175 |
| Age (yr) | 3 | -4 | 10 |
| Sex (female or male) | 37 | -143 | 216 |

**Model 4**

| **Constant and factors** | **Unstandardized coefficient B** | **95% CI** | |
| --- | --- | --- | --- |
|  |  | **Lower limit** | **Upper limit** |
| Constant | -727 | -1304 | -150 |
| Age (yr) | 3 | -5 | 12 |
| Sex (female or male) | 15 | -177 | 207 |
| Physical demands (yes or no) | 40 | -160 | 239 |
| Pain (mm VAS) | 2 | -1 | 6 |
| History of shoulder pain (yes or no) | 45 | -144 | 234 |
| Symptom duration (wk) | -0.3 | -2 | 2 |
| Smoking (yes or no) | 90 | -160 | 341 |

Model 5

| **Constant and factors** | **Unstandardized coefficient B** | **95% CI** | |
| --- | --- | --- | --- |
|  |  | **Lower limit** | **Upper limit** |
| Constant | -388 | -713 | -63 |
| Disability (WORC_1_ADJ_ score) | -0.3 | -1 | 0.2 |
| Pain catastrophizing (PCS score) | 16 | 6 | 27 |

Model 7 (Model 6 was not analysed, see main text of article)

| **Constant and factors** | **Unstandardized coefficient B** | **95% CI** | |
| --- | --- | --- | --- |
|  |  | **Lower limit** | **Upper limit** |
| Constant | -380 | -519 | -241 |
| History of shoulder pain (yes or no) | 61 | -115 | 236 |
| Symptom duration (wk) | -1 | -3 | 1 |

Model 8

| **Constant and factors** | **Unstandardized coefficient B** | **95% CI** | |
| --- | --- | --- | --- |
|  |  | **Lower limit** | **Upper limit** |
| Constant | -513 | -776 | -249 |
| Pain (mm VAS) | 2 | -1 | 5 |
| History of shoulder pain (yes or no) | 60 | -115 | 235 |
| Symptom duration (wk) | -1 | -2 | 1 |

Model 9

| **Constant and factors** | **Unstandardized coefficient B** | **95% CI** | |
| --- | --- | --- | --- |
|  |  | **Lower limit** | **Upper limit** |
| Constant | -596 | -822 | -371 |
| Pain (mm VAS) | 1 | -3 | 4 |
| Pain catastrophizing (PCS score) | 12 | 2 | 22 |
